# [(1,2,5,6-η)-1,5-Cyclo­octa­diene](1-isopropyl-3-methyl­imidazolin-2-yl­idene)(triphenyl­phosphine)iridium(I) tetra­fluorido­borate dichloro­methane solvate

**DOI:** 10.1107/S1600536810031727

**Published:** 2010-08-18

**Authors:** Gary S. Nichol, Daniel Stasiw, Laura J. Anna, Edward Rajaseelan

**Affiliations:** a Department of Chemistry and Biochemistry, The University of Arizona, 1306 E University Boulevard, Tucson, AZ 85721, USA; b Department of Chemistry, Millersville University, Millersville, PA 17551, USA

## Abstract

In the title compound, [Ir(C_8_H_12_)(C_7_H_12_N_2_)(C_18_H_15_P)]BF_4_·CH_2_Cl_2_, the Ir(I) atom has a square-planar conformation with normal bond lengths. One of the phenyl rings, and the solvent dichloro­methane mol­ecule, were refined using separate two part disorder models, each in an approximately 1:1 ratio.

## Related literature

For structure and dynamics of related *N*-heterocyclic carbene iridium complexes, see: Chianese *et al.* (2003 ▶); Herrmann *et al.* (2006 ▶); Köcher & Herrmann (1997 ▶); Nichol *et al.* (2009 ▶). For the isotypic Rh analogue, see: Nichol *et al.* (2010 ▶). For catalytic properties of these complexes, see: Albrecht *et al.* (2002 ▶); Frey *et al.* (2006 ▶); Gnanamgari *et al.* (2007 ▶); Voutchkova *et al.* (2008 ▶).
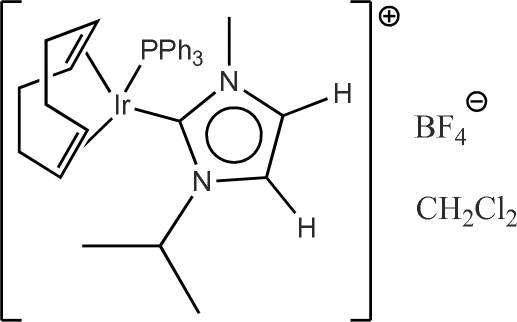


## Experimental

### 

#### Crystal data

[Ir(C_8_H_12_)(C_7_H_12_N_2_)(C_18_H_15_P)]BF_4_·CH_2_Cl_2_
*M* *_r_* = 858.57
Monoclinic, 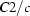

*a* = 36.3039 (16) Å
*b* = 10.4913 (5) Å
*c* = 18.3924 (8) Å
β = 103.452 (2)°
*V* = 6813.0 (5) Å^3^
*Z* = 8
Mo *K*α radiation
μ = 4.17 mm^−1^
*T* = 100 K
0.30 × 0.14 × 0.11 mm

#### Data collection

Bruker Kappa APEXII DUO CCD diffractometer
Absorption correction: numerical (*SADABS*; Sheldrick, 1996 ▶) *T*_min_ = 0.370, *T*_max_ = 0.652
72041 measured reflections
7834 independent reflections
6800 reflections with *I* > 2σ(*I*)
*R*_int_ = 0.033

#### Refinement

*R*[*F* ^2^ > 2σ(*F* ^2^)] = 0.021
*wR*(*F* ^2^) = 0.073
*S* = 1.04
7834 reflections
460 parameters
84 restraints
H-atom parameters constrained
Δρ_max_ = 1.31 e Å^−3^
Δρ_min_ = −0.94 e Å^−3^

### 

Data collection: *APEX2* (Bruker, 2007 ▶); cell refinement: *SAINT* (Bruker, 2007 ▶); data reduction: *SAINT*; program(s) used to solve structure: *SIR97* (Altomare *et al.*, 1999 ▶); program(s) used to refine structure: *SHELXTL* (Sheldrick, 2008 ▶); molecular graphics: *ORTEP-3 for Windows* (Farrugia, 1997 ▶); software used to prepare material for publication: *SHELXTL* and *publCIF* (Westrip, 2010 ▶).

## Supplementary Material

Crystal structure: contains datablocks I, global. DOI: 10.1107/S1600536810031727/ng5007sup1.cif

Structure factors: contains datablocks I. DOI: 10.1107/S1600536810031727/ng5007Isup2.hkl

Additional supplementary materials:  crystallographic information; 3D view; checkCIF report

## Figures and Tables

**Fig. 1.**
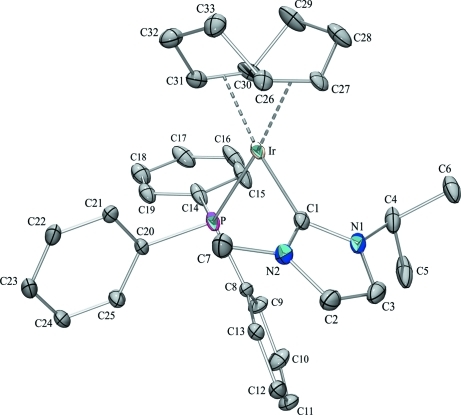
The structure of the Ir complex in (I), with displacement ellipsoids at the 30% probability level and hydrogen atoms omitted.

## supplementary crystallographic information

### Comment

The title compound, (I), was prepared as part of our ongoing research on Rh and Ir-containing N-heterocyclic carbene complexes (Nichol *et al.*, 2009). The Ir center has an expected square planar conformation and bond distances are unexceptional. One of the phenyl rings, and the solvent dichloromethane molecule, were refined using separate two part disorder models, each in an approximately 1:1 ratio. The structure of the isomorphous Rh analogue has also been reported (Nichol *et al.*, 2010).

### Experimental

The title compound, (I), was synthesized by the reaction of [Ir(cod)(C_7_H_12_N_2_)Cl](0.369 mg, 0.80 mmol) and triphenylphosphene (211 mg, 0.80 mmol) in CH_2_Cl_2_ (8 ml) with silver tetrafluoroborate (157 m, 0.80 mmol) for 1.5 h. The mixture was filtered through Celite to remove silver chloride and the solvent was removed under reduced pressure. Crystals of the resulting solid of the title compound were obtained by slow diffusion of pentane into dichloromethane solution of the compound.

### Refinement

One of the phenyl rings, and the solvent dichloromethane molecule, were refined using separate two part disorder models, each in an approximately 1:1 ratio. Bond distance similarity restraints were applied to the dichloromethane molecule, and approximate isotropic restraints were used on the ellipsoids of the disordered C atoms of both the dichloromethane molecule and the phenyl ring. The largest residual peak is approximately 0.76Å from Ir. H atoms were first located in a difference map and then refined using *U*_iso_H = 1.5*U*_eq_C for methyl groups and *U*_iso_H = 1.2*U*_eq_C for all others and constrained C–H distances.

### Crystal data

**Table:**

[Ir(C_8_H_12_)(C_7_H_12_N_2_)(C_18_H_15_P)]BF_4_·CH_2_Cl_2_	*F*(000) = 3408
*M**_r_* = 858.57	*D*_x_ = 1.674 Mg m^−^^3^
Monoclinic, *C*2/*c*	Mo *K*α radiation, λ = 0.71073 Å
Hall symbol: -C 2yc	Cell parameters from 9491 reflections
*a* = 36.3039 (16) Å	θ = 2.4–28.3°
*b* = 10.4913 (5) Å	µ = 4.17 mm^−^^1^
*c* = 18.3924 (8) Å	*T* = 100 K
β = 103.452 (2)°	Prism, red
*V* = 6813.0 (5) Å^3^	0.30 × 0.14 × 0.11 mm
*Z* = 8	

### Data collection

**Table:**

Bruker Kappa APEXII DUO CCD diffractometer	7834 independent reflections
Radiation source: fine-focus sealed tube with Miracol optics	6800 reflections with *I* > 2σ(*I*)
graphite	*R*_int_ = 0.033
φ and ω scans	θ_max_ = 27.5°, θ_min_ = 2.0°
Absorption correction: numerical (*SADABS*; Sheldrick, 1996)	*h* = −47→47
*T*_min_ = 0.370, *T*_max_ = 0.652	*k* = −13→13
72041 measured reflections	*l* = −23→23

### Refinement

**Table:**

Refinement on *F*^2^	Primary atom site location: structure-invariant direct methods
Least-squares matrix: full	Secondary atom site location: difference Fourier map
*R*[*F*^2^ > 2σ(*F*^2^)] = 0.021	Hydrogen site location: inferred from neighbouring sites
*wR*(*F*^2^) = 0.073	H-atom parameters constrained
*S* = 1.04	*w* = 1/[σ^2^(*F*_o_^2^) + (0.050*P*)^2^ + 5.*P*] where *P* = (*F*_o_^2^ + 2*F*_c_^2^)/3
7834 reflections	(Δ/σ)_max_ = 0.011
460 parameters	Δρ_max_ = 1.31 e Å^−^^3^
84 restraints	Δρ_min_ = −0.94 e Å^−^^3^

### Special details

**Table:**

Geometry. All e.s.d.'s (except the e.s.d. in the dihedral angle between two l.s. planes) are estimated using the full covariance matrix. The cell e.s.d.'s are taken into account individually in the estimation of e.s.d.'s in distances, angles and torsion angles; correlations between e.s.d.'s in cell parameters are only used when they are defined by crystal symmetry. An approximate (isotropic) treatment of cell e.s.d.'s is used for estimating e.s.d.'s involving l.s. planes.
Refinement. Refinement of *F*^2^ against ALL reflections. The weighted *R*-factor *wR* and goodness of fit *S* are based on *F*^2^, conventional *R*-factors *R* are based on *F*, with *F* set to zero for negative *F*^2^. The threshold expression of *F*^2^ > σ(*F*^2^) is used only for calculating *R*-factors(gt) *etc*. and is not relevant to the choice of reflections for refinement. *R*-factors based on *F*^2^ are statistically about twice as large as those based on *F*, and *R*- factors based on ALL data will be even larger.

### Fractional atomic coordinates and isotropic or equivalent isotropic displacement parameters (Å^2^)

**Table:**

	*x*	*y*	*z*	*U*_iso_*/*U*_eq_	Occ. (<1)
Ir	0.668273 (3)	0.510395 (9)	0.563854 (6)	0.02420 (5)	
Cl1	0.54347 (6)	0.9363 (4)	0.70029 (17)	0.0471 (7)	0.511 (7)
Cl2	0.57718 (16)	1.1103 (5)	0.6136 (5)	0.137 (2)	0.511 (7)
Cl1'	0.54270 (8)	1.0031 (6)	0.71548 (15)	0.0579 (11)	0.489 (7)
Cl2'	0.57231 (13)	1.0807 (4)	0.58942 (14)	0.0553 (9)	0.489 (7)
P	0.60271 (2)	0.52250 (7)	0.53810 (5)	0.02869 (16)	
F1	0.63097 (6)	1.1774 (2)	0.28542 (14)	0.0718 (7)	
F2	0.68831 (7)	1.2144 (3)	0.26529 (15)	0.0974 (10)	
F3	0.68298 (6)	1.1205 (3)	0.37193 (13)	0.0728 (7)	
F4	0.66639 (8)	1.0113 (2)	0.2656 (2)	0.0865 (10)	
N1	0.67636 (6)	0.7514 (2)	0.47676 (15)	0.0373 (6)	
N2	0.67143 (7)	0.5884 (2)	0.40379 (14)	0.0356 (6)	
B	0.66725 (11)	1.1321 (5)	0.2973 (3)	0.0557 (11)	
C1	0.67157 (7)	0.6230 (3)	0.47509 (17)	0.0318 (6)	
C2	0.67518 (8)	0.6943 (3)	0.3618 (2)	0.0455 (8)	
H2	0.6756	0.6948	0.3104	0.055*	
C3	0.67807 (8)	0.7960 (3)	0.4063 (2)	0.0488 (9)	
H3	0.6808	0.8822	0.3926	0.059*	
C4	0.67913 (8)	0.8306 (3)	0.5440 (2)	0.0456 (8)	
H4	0.6763	0.7737	0.5860	0.055*	
C5	0.64780 (9)	0.9295 (3)	0.5322 (3)	0.0681 (13)	
H5A	0.6232	0.8869	0.5172	0.102*	
H5B	0.6508	0.9890	0.4929	0.102*	
H5C	0.6492	0.9765	0.5788	0.102*	
C6	0.71784 (8)	0.8956 (3)	0.5664 (2)	0.0525 (9)	
H6A	0.7378	0.8324	0.5672	0.079*	
H6B	0.7210	0.9335	0.6162	0.079*	
H6C	0.7195	0.9625	0.5301	0.079*	
C7	0.66684 (10)	0.4574 (3)	0.37577 (19)	0.0403 (7)	
H7A	0.6580	0.4034	0.4118	0.060*	
H7B	0.6912	0.4254	0.3691	0.060*	
H7C	0.6482	0.4556	0.3277	0.060*	
C8	0.57817 (16)	0.6605 (5)	0.4981 (5)	0.0241 (16)	0.556 (18)
C9	0.55354 (16)	0.7332 (5)	0.5287 (5)	0.0363 (17)	0.556 (18)
H9	0.5505	0.7149	0.5776	0.044*	0.556 (18)
C10	0.53335 (13)	0.8325 (4)	0.4879 (7)	0.047 (2)	0.556 (18)
H10	0.5165	0.8822	0.5088	0.057*	0.556 (18)
C11	0.53780 (13)	0.8592 (4)	0.4164 (7)	0.041 (2)	0.556 (18)
H11	0.5240	0.9272	0.3885	0.049*	0.556 (18)
C12	0.56243 (15)	0.7866 (6)	0.3857 (5)	0.041 (2)	0.556 (18)
H12	0.5655	0.8049	0.3369	0.049*	0.556 (18)
C13	0.58261 (15)	0.6873 (5)	0.4266 (5)	0.0284 (17)	0.556 (18)
H13	0.5994	0.6376	0.4056	0.034*	0.556 (18)
C8'	0.5794 (2)	0.6616 (5)	0.4725 (5)	0.021 (2)	0.444 (18)
C9'	0.55502 (19)	0.7448 (6)	0.4970 (6)	0.035 (2)	0.444 (18)
H9'	0.5509	0.7363	0.5459	0.042*	0.444 (18)
C10'	0.53665 (19)	0.8404 (6)	0.4499 (7)	0.040 (3)	0.444 (18)
H10'	0.5200	0.8973	0.4666	0.048*	0.444 (18)
C11'	0.5426 (2)	0.8528 (5)	0.3783 (7)	0.042 (3)	0.444 (18)
H11'	0.5301	0.9182	0.3461	0.050*	0.444 (18)
C12'	0.5670 (2)	0.7697 (6)	0.3537 (6)	0.038 (2)	0.444 (18)
H12'	0.5711	0.7781	0.3048	0.046*	0.444 (18)
C13'	0.5854 (2)	0.6741 (6)	0.4008 (5)	0.0260 (19)	0.444 (18)
H13'	0.6020	0.6172	0.3841	0.031*	0.444 (18)
C14	0.58120 (8)	0.5100 (3)	0.6183 (2)	0.0362 (7)	
C15	0.59156 (8)	0.5988 (3)	0.6765 (2)	0.0471 (9)	
H15	0.6098	0.6628	0.6740	0.057*	
C16	0.57541 (8)	0.5937 (4)	0.7375 (2)	0.0510 (9)	
H16	0.5820	0.6558	0.7759	0.061*	
C17	0.54978 (10)	0.4992 (3)	0.7431 (2)	0.0496 (10)	
H17	0.5391	0.4951	0.7856	0.060*	
C18	0.53965 (8)	0.4100 (3)	0.68616 (18)	0.0429 (7)	
H18	0.5220	0.3446	0.6897	0.052*	
C19	0.55513 (7)	0.4160 (3)	0.62424 (17)	0.0351 (6)	
H19	0.5478	0.3550	0.5853	0.042*	
C20	0.58018 (7)	0.3948 (2)	0.47589 (14)	0.0255 (5)	
C21	0.60049 (8)	0.2880 (2)	0.46441 (14)	0.0265 (5)	
H21	0.6264	0.2804	0.4895	0.032*	
C22	0.58319 (9)	0.1918 (3)	0.41636 (16)	0.0344 (6)	
H22	0.5973	0.1191	0.4082	0.041*	
C23	0.54526 (10)	0.2028 (3)	0.38053 (17)	0.0412 (7)	
H23	0.5333	0.1370	0.3481	0.049*	
C24	0.52498 (9)	0.3086 (3)	0.39171 (18)	0.0432 (8)	
H24	0.4991	0.3156	0.3665	0.052*	
C25	0.54182 (8)	0.4050 (3)	0.43925 (17)	0.0360 (6)	
H25	0.5275	0.4775	0.4470	0.043*	
C26	0.72262 (7)	0.4198 (3)	0.56404 (17)	0.0310 (6)	
H26	0.7287	0.4180	0.5137	0.037*	
C27	0.73017 (8)	0.5364 (3)	0.60018 (18)	0.0333 (6)	
H27	0.7404	0.6019	0.5707	0.040*	
C28	0.74038 (8)	0.5601 (4)	0.68319 (19)	0.0484 (8)	
H28A	0.7677	0.5421	0.7026	0.058*	
H28B	0.7362	0.6513	0.6925	0.058*	
C29	0.71816 (11)	0.4810 (4)	0.7254 (2)	0.0515 (10)	
H29A	0.7177	0.5245	0.7729	0.062*	
H29B	0.7310	0.3980	0.7379	0.062*	
C30	0.67744 (8)	0.4573 (4)	0.68168 (17)	0.0398 (7)	
H30	0.6578	0.4914	0.7067	0.048*	
C31	0.66613 (8)	0.3492 (3)	0.63912 (15)	0.0335 (6)	
H31	0.6399	0.3201	0.6391	0.040*	
C32	0.69224 (8)	0.2436 (3)	0.62553 (18)	0.0413 (7)	
H32A	0.6999	0.1918	0.6716	0.050*	
H32B	0.6781	0.1874	0.5854	0.050*	
C33	0.72751 (9)	0.2912 (3)	0.60362 (19)	0.0421 (7)	
H33A	0.7353	0.2275	0.5704	0.050*	
H33B	0.7482	0.2980	0.6492	0.050*	
C34	0.58431 (16)	0.9833 (6)	0.6744 (3)	0.0401 (17)	0.511 (7)
H34A	0.6037	1.0068	0.7198	0.048*	0.511 (7)
H34B	0.5942	0.9105	0.6505	0.048*	0.511 (7)
C34'	0.58224 (16)	1.0511 (6)	0.6851 (3)	0.081 (3)*	0.489 (7)
H34C	0.6019	0.9840	0.6974	0.097*	0.489 (7)
H34D	0.5926	1.1295	0.7122	0.097*	0.489 (7)

### Atomic displacement parameters (Å^2^)

**Table:**

	*U*^11^	*U*^22^	*U*^33^	*U*^12^	*U*^13^	*U*^23^
Ir	0.01353 (7)	0.02279 (7)	0.03367 (8)	0.00024 (3)	0.00019 (5)	−0.00506 (4)
Cl1	0.0276 (8)	0.0522 (16)	0.0600 (13)	−0.0038 (8)	0.0068 (8)	−0.0006 (11)
Cl2	0.082 (3)	0.064 (2)	0.293 (7)	0.033 (2)	0.100 (4)	0.095 (4)
Cl1'	0.0481 (13)	0.087 (3)	0.0383 (11)	−0.0257 (13)	0.0087 (9)	0.0051 (12)
Cl2'	0.0796 (19)	0.0548 (18)	0.0410 (14)	0.0281 (14)	0.0329 (10)	0.0115 (9)
P	0.0143 (3)	0.0221 (3)	0.0463 (4)	−0.0004 (2)	0.0001 (3)	−0.0069 (3)
F1	0.0373 (11)	0.0748 (16)	0.1002 (18)	0.0227 (11)	0.0097 (11)	0.0172 (14)
F2	0.0634 (16)	0.135 (3)	0.0935 (19)	−0.0184 (16)	0.0181 (14)	0.0535 (18)
F3	0.0535 (13)	0.0842 (18)	0.0747 (15)	0.0031 (12)	0.0025 (11)	0.0362 (13)
F4	0.0402 (15)	0.085 (2)	0.124 (3)	0.0264 (11)	−0.0033 (15)	−0.0120 (15)
N1	0.0173 (11)	0.0250 (12)	0.0645 (17)	−0.0015 (9)	−0.0010 (10)	0.0063 (11)
N2	0.0264 (12)	0.0322 (13)	0.0443 (14)	−0.0048 (10)	0.0006 (10)	0.0080 (11)
B	0.0281 (19)	0.069 (3)	0.069 (3)	0.0096 (18)	0.0080 (18)	0.022 (2)
C1	0.0162 (12)	0.0253 (13)	0.0496 (17)	−0.0019 (10)	−0.0013 (11)	0.0031 (12)
C2	0.0313 (16)	0.0437 (18)	0.056 (2)	−0.0060 (13)	−0.0008 (14)	0.0207 (15)
C3	0.0252 (15)	0.0365 (17)	0.079 (2)	−0.0033 (13)	−0.0002 (15)	0.0248 (17)
C4	0.0248 (14)	0.0228 (14)	0.087 (2)	−0.0024 (11)	0.0078 (15)	−0.0061 (15)
C5	0.0224 (15)	0.0287 (17)	0.146 (4)	−0.0007 (13)	0.0055 (19)	−0.014 (2)
C6	0.0221 (15)	0.0325 (16)	0.096 (3)	−0.0015 (12)	−0.0004 (16)	−0.0139 (17)
C7	0.0423 (18)	0.0382 (16)	0.0394 (17)	−0.0084 (15)	0.0073 (13)	0.0021 (14)
C8	0.017 (2)	0.024 (3)	0.029 (4)	−0.0035 (19)	0.000 (2)	0.006 (2)
C9	0.032 (3)	0.028 (3)	0.047 (4)	0.013 (2)	0.006 (3)	0.010 (3)
C10	0.048 (3)	0.033 (3)	0.063 (6)	0.015 (3)	0.017 (3)	0.013 (3)
C11	0.039 (3)	0.032 (3)	0.047 (6)	0.007 (2)	0.000 (3)	0.016 (3)
C12	0.036 (3)	0.039 (4)	0.041 (5)	−0.010 (3)	−0.004 (3)	0.014 (3)
C13	0.022 (3)	0.030 (3)	0.030 (4)	−0.002 (2)	0.001 (3)	0.001 (3)
C8'	0.018 (3)	0.020 (3)	0.024 (5)	−0.001 (2)	0.004 (3)	−0.001 (3)
C9'	0.035 (4)	0.029 (4)	0.035 (5)	0.005 (3)	0.000 (3)	−0.004 (3)
C10'	0.043 (4)	0.033 (4)	0.040 (6)	0.020 (3)	0.003 (4)	0.003 (4)
C11'	0.051 (4)	0.023 (3)	0.042 (6)	0.011 (3)	−0.007 (4)	0.004 (3)
C12'	0.041 (4)	0.031 (4)	0.036 (5)	−0.002 (3)	−0.003 (3)	0.011 (3)
C13'	0.027 (3)	0.024 (3)	0.025 (4)	−0.003 (2)	0.003 (3)	0.005 (3)
C14	0.0134 (13)	0.0382 (16)	0.055 (2)	0.0014 (10)	0.0038 (13)	−0.0189 (13)
C15	0.0152 (13)	0.055 (2)	0.070 (2)	−0.0043 (12)	0.0079 (13)	−0.0321 (17)
C16	0.0195 (14)	0.072 (2)	0.058 (2)	0.0024 (14)	0.0016 (13)	−0.0355 (18)
C17	0.0231 (17)	0.073 (3)	0.051 (2)	0.0063 (13)	0.0047 (15)	−0.0193 (16)
C18	0.0244 (14)	0.0518 (19)	0.0511 (19)	−0.0001 (13)	0.0059 (13)	−0.0088 (15)
C19	0.0183 (12)	0.0370 (15)	0.0476 (17)	0.0010 (11)	0.0024 (11)	−0.0113 (13)
C20	0.0203 (12)	0.0231 (12)	0.0294 (13)	−0.0042 (9)	−0.0015 (10)	0.0005 (10)
C21	0.0267 (13)	0.0236 (12)	0.0275 (13)	−0.0025 (10)	0.0026 (10)	0.0048 (10)
C22	0.0464 (17)	0.0208 (13)	0.0341 (15)	−0.0040 (12)	0.0056 (13)	0.0003 (11)
C23	0.0508 (19)	0.0295 (15)	0.0346 (16)	−0.0129 (13)	−0.0077 (14)	0.0013 (12)
C24	0.0323 (16)	0.0346 (16)	0.0497 (18)	−0.0103 (12)	−0.0171 (13)	0.0066 (13)
C25	0.0242 (14)	0.0280 (14)	0.0486 (17)	−0.0001 (11)	−0.0062 (12)	0.0005 (12)
C26	0.0177 (12)	0.0316 (14)	0.0445 (16)	0.0034 (10)	0.0090 (11)	−0.0012 (12)
C27	0.0112 (12)	0.0369 (15)	0.0479 (18)	0.0001 (11)	−0.0014 (11)	−0.0046 (14)
C28	0.0222 (14)	0.065 (2)	0.0515 (19)	0.0020 (15)	−0.0043 (13)	−0.0185 (17)
C29	0.0306 (18)	0.084 (3)	0.0360 (18)	−0.0043 (16)	−0.0005 (14)	−0.0196 (16)
C30	0.0221 (14)	0.066 (2)	0.0291 (15)	0.0058 (14)	0.0026 (11)	−0.0068 (15)
C31	0.0264 (14)	0.0429 (16)	0.0302 (14)	0.0021 (12)	0.0041 (11)	0.0076 (12)
C32	0.0359 (16)	0.0404 (17)	0.0467 (17)	0.0094 (13)	0.0074 (13)	0.0117 (14)
C33	0.0360 (16)	0.0327 (16)	0.058 (2)	0.0082 (13)	0.0126 (14)	0.0022 (14)
C34	0.034 (3)	0.026 (3)	0.066 (4)	0.004 (2)	0.022 (3)	−0.002 (3)

### Geometric parameters (Å, °)

**Table:**

Ir—P	2.3196 (7)	C10'—H10'	0.950
Ir—C1	2.041 (3)	C10'—C11'	1.390
Ir—C26	2.189 (3)	C11'—H11'	0.950
Ir—C27	2.207 (3)	C11'—C12'	1.390
Ir—C30	2.187 (3)	C12'—H12'	0.950
Ir—C31	2.198 (3)	C12'—C13'	1.390
Cl1—C34	1.731 (5)	C13'—H13'	0.950
Cl2—C34	1.719 (6)	C14—C15	1.404 (4)
Cl1'—C34'	1.732 (5)	C14—C19	1.389 (4)
Cl2'—C34'	1.740 (5)	C15—H15	0.950
P—C8	1.769 (4)	C15—C16	1.382 (5)
P—C8'	1.957 (6)	C16—H16	0.950
P—C14	1.827 (4)	C16—C17	1.380 (5)
P—C20	1.827 (3)	C17—H17	0.950
F1—B	1.369 (4)	C17—C18	1.389 (5)
F2—B	1.373 (5)	C18—H18	0.950
F3—B	1.364 (5)	C18—C19	1.384 (4)
F4—B	1.393 (6)	C19—H19	0.950
N1—C1	1.358 (3)	C20—C21	1.384 (4)
N1—C3	1.392 (4)	C20—C25	1.403 (4)
N1—C4	1.474 (4)	C21—H21	0.950
N2—C1	1.360 (4)	C21—C22	1.391 (4)
N2—C2	1.377 (4)	C22—H22	0.950
N2—C7	1.464 (4)	C22—C23	1.386 (4)
C2—H2	0.950	C23—H23	0.950
C2—C3	1.334 (5)	C23—C24	1.373 (5)
C3—H3	0.950	C24—H24	0.950
C4—H4	1.000	C24—C25	1.383 (4)
C4—C5	1.517 (4)	C25—H25	0.950
C4—C6	1.529 (4)	C26—H26	1.000
C5—H5A	0.980	C26—C27	1.388 (4)
C5—H5B	0.980	C26—C33	1.524 (4)
C5—H5C	0.980	C27—H27	1.000
C6—H6A	0.980	C27—C28	1.506 (5)
C6—H6B	0.980	C28—H28A	0.990
C6—H6C	0.980	C28—H28B	0.990
C7—H7A	0.980	C28—C29	1.494 (6)
C7—H7B	0.980	C29—H29A	0.990
C7—H7C	0.980	C29—H29B	0.990
C8—C9	1.390	C29—C30	1.530 (5)
C8—C13	1.390	C30—H30	1.000
C9—H9	0.950	C30—C31	1.384 (4)
C9—C10	1.390	C31—H31	1.000
C10—H10	0.950	C31—C32	1.516 (4)
C10—C11	1.390	C32—H32A	0.990
C11—H11	0.950	C32—H32B	0.990
C11—C12	1.390	C32—C33	1.514 (4)
C12—H12	0.950	C33—H33A	0.990
C12—C13	1.390	C33—H33B	0.990
C13—H13	0.950	C34—H34A	0.990
C8'—C9'	1.390	C34—H34B	0.990
C8'—C13'	1.390	C34'—H34C	0.990
C9'—H9'	0.950	C34'—H34D	0.990
C9'—C10'	1.390		
			
P—Ir—C1	93.05 (7)	C11'—C12'—C13'	120.0
P—Ir—C26	154.91 (8)	H12'—C12'—C13'	120.0
P—Ir—C27	168.25 (9)	C8'—C13'—C12'	120.0
P—Ir—C30	97.40 (8)	C8'—C13'—H13'	120.0
P—Ir—C31	89.18 (8)	C12'—C13'—H13'	120.0
C1—Ir—C26	91.82 (11)	P—C14—C15	118.7 (2)
C1—Ir—C27	85.73 (11)	P—C14—C19	122.8 (2)
C1—Ir—C30	156.42 (12)	C15—C14—C19	118.5 (3)
C1—Ir—C31	165.05 (11)	C14—C15—H15	119.9
C26—Ir—C27	36.81 (11)	C14—C15—C16	120.3 (3)
C26—Ir—C30	87.62 (11)	H15—C15—C16	119.9
C26—Ir—C31	80.16 (11)	C15—C16—H16	119.7
C27—Ir—C30	79.75 (12)	C15—C16—C17	120.6 (3)
C27—Ir—C31	95.00 (12)	H16—C16—C17	119.7
C30—Ir—C31	36.81 (12)	C16—C17—H17	120.3
Ir—P—C8	121.6 (2)	C16—C17—C18	119.5 (4)
Ir—P—C8'	116.3 (3)	H17—C17—C18	120.3
Ir—P—C14	116.25 (11)	C17—C18—H18	119.9
Ir—P—C20	112.12 (9)	C17—C18—C19	120.2 (3)
C8—P—C8'	13.7 (2)	H18—C18—C19	119.9
C8—P—C14	97.2 (3)	C14—C19—C18	120.8 (3)
C8—P—C20	103.3 (3)	C14—C19—H19	119.6
C8'—P—C14	110.3 (3)	C18—C19—H19	119.6
C8'—P—C20	95.4 (3)	P—C20—C21	120.81 (19)
C14—P—C20	103.87 (13)	P—C20—C25	119.7 (2)
C1—N1—C3	110.2 (3)	C21—C20—C25	119.5 (2)
C1—N1—C4	124.2 (3)	C20—C21—H21	119.8
C3—N1—C4	125.6 (3)	C20—C21—C22	120.4 (2)
C1—N2—C2	110.3 (3)	H21—C21—C22	119.8
C1—N2—C7	124.4 (2)	C21—C22—H22	120.2
C2—N2—C7	125.3 (3)	C21—C22—C23	119.6 (3)
F1—B—F2	108.9 (3)	H22—C22—C23	120.2
F1—B—F3	110.7 (4)	C22—C23—H23	119.9
F1—B—F4	108.8 (4)	C22—C23—C24	120.3 (3)
F2—B—F3	110.1 (4)	H23—C23—C24	119.9
F2—B—F4	110.7 (4)	C23—C24—H24	119.6
F3—B—F4	107.7 (4)	C23—C24—C25	120.8 (3)
Ir—C1—N1	125.9 (2)	H24—C24—C25	119.6
Ir—C1—N2	129.0 (2)	C20—C25—C24	119.4 (3)
N1—C1—N2	105.0 (3)	C20—C25—H25	120.3
N2—C2—H2	126.1	C24—C25—H25	120.3
N2—C2—C3	107.8 (3)	Ir—C26—H26	113.5
H2—C2—C3	126.1	Ir—C26—C27	72.31 (15)
N1—C3—C2	106.7 (3)	Ir—C26—C33	112.95 (18)
N1—C3—H3	126.6	H26—C26—C27	113.5
C2—C3—H3	126.6	H26—C26—C33	113.5
N1—C4—H4	108.1	C27—C26—C33	124.3 (3)
N1—C4—C5	111.3 (3)	Ir—C27—C26	70.88 (15)
N1—C4—C6	110.8 (3)	Ir—C27—H27	113.7
H4—C4—C5	108.1	Ir—C27—C28	108.7 (2)
H4—C4—C6	108.1	C26—C27—H27	113.7
C5—C4—C6	110.2 (3)	C26—C27—C28	127.3 (3)
C4—C5—H5A	109.5	H27—C27—C28	113.7
C4—C5—H5B	109.5	C27—C28—H28A	108.9
C4—C5—H5C	109.5	C27—C28—H28B	108.9
H5A—C5—H5B	109.5	C27—C28—C29	113.6 (3)
H5A—C5—H5C	109.5	H28A—C28—H28B	107.7
H5B—C5—H5C	109.5	H28A—C28—C29	108.9
C4—C6—H6A	109.5	H28B—C28—C29	108.9
C4—C6—H6B	109.5	C28—C29—H29A	109.0
C4—C6—H6C	109.5	C28—C29—H29B	109.0
H6A—C6—H6B	109.5	C28—C29—C30	112.9 (3)
H6A—C6—H6C	109.5	H29A—C29—H29B	107.8
H6B—C6—H6C	109.5	H29A—C29—C30	109.0
N2—C7—H7A	109.5	H29B—C29—C30	109.0
N2—C7—H7B	109.5	Ir—C30—C29	112.7 (2)
N2—C7—H7C	109.5	Ir—C30—H30	113.8
H7A—C7—H7B	109.5	Ir—C30—C31	72.04 (17)
H7A—C7—H7C	109.5	C29—C30—H30	113.8
H7B—C7—H7C	109.5	C29—C30—C31	123.6 (3)
P—C8—C9	126.1 (3)	H30—C30—C31	113.8
P—C8—C13	113.6 (3)	Ir—C31—C30	71.15 (19)
C9—C8—C13	120.0	Ir—C31—H31	114.1
C8—C9—H9	120.0	Ir—C31—C32	110.07 (18)
C8—C9—C10	120.0	C30—C31—H31	114.1
H9—C9—C10	120.0	C30—C31—C32	125.2 (3)
C9—C10—H10	120.0	H31—C31—C32	114.1
C9—C10—C11	120.0	C31—C32—H32A	108.8
H10—C10—C11	120.0	C31—C32—H32B	108.8
C10—C11—H11	120.0	C31—C32—C33	113.8 (3)
C10—C11—C12	120.0	H32A—C32—H32B	107.7
H11—C11—C12	120.0	H32A—C32—C33	108.8
C11—C12—H12	120.0	H32B—C32—C33	108.8
C11—C12—C13	120.0	C26—C33—C32	114.0 (2)
H12—C12—C13	120.0	C26—C33—H33A	108.7
C8—C13—C12	120.0	C26—C33—H33B	108.7
C8—C13—H13	120.0	C32—C33—H33A	108.7
C12—C13—H13	120.0	C32—C33—H33B	108.7
P—C8'—C9'	119.0 (3)	H33A—C33—H33B	107.6
P—C8'—C13'	120.9 (3)	Cl1—C34—Cl2	113.0 (4)
C9'—C8'—C13'	120.0	Cl1—C34—H34A	109.0
C8'—C9'—H9'	120.0	Cl1—C34—H34B	109.0
C8'—C9'—C10'	120.0	Cl2—C34—H34A	109.0
H9'—C9'—C10'	120.0	Cl2—C34—H34B	109.0
C9'—C10'—H10'	120.0	H34A—C34—H34B	107.8
C9'—C10'—C11'	120.0	Cl1'—C34'—Cl2'	112.7 (4)
H10'—C10'—C11'	120.0	Cl1'—C34'—H34C	109.1
C10'—C11'—H11'	120.0	Cl1'—C34'—H34D	109.1
C10'—C11'—C12'	120.0	Cl2'—C34'—H34C	109.1
H11'—C11'—C12'	120.0	Cl2'—C34'—H34D	109.1
C11'—C12'—H12'	120.0	H34C—C34'—H34D	107.8
			
C1—Ir—P—C8	−32.6 (3)	C8—P—C14—C19	108.2 (3)
C1—Ir—P—C8'	−18.0 (3)	C8'—P—C14—C15	−76.7 (4)
C1—Ir—P—C14	−150.47 (13)	C8'—P—C14—C19	103.6 (4)
C1—Ir—P—C20	90.25 (12)	C20—P—C14—C15	−177.9 (3)
C26—Ir—P—C8	−133.5 (4)	C20—P—C14—C19	2.4 (3)
C26—Ir—P—C8'	−118.9 (3)	P—C14—C15—C16	178.9 (3)
C26—Ir—P—C14	108.6 (2)	C19—C14—C15—C16	−1.4 (5)
C26—Ir—P—C20	−10.6 (2)	C14—C15—C16—C17	1.9 (5)
C27—Ir—P—C8	51.2 (6)	C15—C16—C17—C18	−1.2 (5)
C27—Ir—P—C8'	65.7 (5)	C16—C17—C18—C19	−0.1 (5)
C27—Ir—P—C14	−66.7 (5)	C17—C18—C19—C14	0.6 (5)
C27—Ir—P—C20	174.0 (4)	P—C14—C19—C18	179.8 (2)
C30—Ir—P—C8	126.3 (3)	C15—C14—C19—C18	0.1 (5)
C30—Ir—P—C8'	140.8 (3)	Ir—P—C20—C21	16.3 (2)
C30—Ir—P—C14	8.35 (14)	Ir—P—C20—C25	−163.7 (2)
C30—Ir—P—C20	−110.93 (14)	C8—P—C20—C21	148.9 (3)
C31—Ir—P—C8	162.2 (3)	C8—P—C20—C25	−31.1 (4)
C31—Ir—P—C8'	176.7 (3)	C8'—P—C20—C21	137.6 (3)
C31—Ir—P—C14	44.32 (13)	C8'—P—C20—C25	−42.4 (4)
C31—Ir—P—C20	−74.96 (12)	C14—P—C20—C21	−110.0 (2)
C3—N1—C1—Ir	178.82 (19)	C14—P—C20—C25	70.0 (3)
C3—N1—C1—N2	1.7 (3)	P—C20—C21—C22	−179.4 (2)
C4—N1—C1—Ir	−1.4 (4)	C25—C20—C21—C22	0.6 (4)
C4—N1—C1—N2	−178.5 (2)	C20—C21—C22—C23	−0.7 (4)
C2—N2—C1—Ir	−178.4 (2)	C21—C22—C23—C24	0.7 (5)
C2—N2—C1—N1	−1.4 (3)	C22—C23—C24—C25	−0.6 (5)
C7—N2—C1—Ir	3.2 (4)	C23—C24—C25—C20	0.6 (5)
C7—N2—C1—N1	−179.8 (2)	P—C20—C25—C24	179.5 (2)
P—Ir—C1—N1	92.8 (2)	C21—C20—C25—C24	−0.5 (4)
P—Ir—C1—N2	−90.8 (2)	P—Ir—C26—C27	−178.43 (16)
C26—Ir—C1—N1	−111.9 (2)	P—Ir—C26—C33	−58.0 (3)
C26—Ir—C1—N2	64.6 (2)	C1—Ir—C26—C27	80.4 (2)
C27—Ir—C1—N1	−75.5 (2)	C1—Ir—C26—C33	−159.1 (2)
C27—Ir—C1—N2	100.9 (2)	C27—Ir—C26—C33	120.5 (3)
C30—Ir—C1—N1	−23.6 (4)	C30—Ir—C26—C27	−76.0 (2)
C30—Ir—C1—N2	152.8 (3)	C30—Ir—C26—C33	44.4 (2)
C31—Ir—C1—N1	−168.9 (3)	C31—Ir—C26—C27	−112.3 (2)
C31—Ir—C1—N2	7.5 (5)	C31—Ir—C26—C33	8.2 (2)
C1—N2—C2—C3	0.6 (3)	Ir—C26—C27—C28	99.3 (3)
C7—N2—C2—C3	178.9 (3)	C33—C26—C27—Ir	−106.1 (3)
N2—C2—C3—N1	0.4 (3)	C33—C26—C27—C28	−6.8 (4)
C1—N1—C3—C2	−1.4 (3)	P—Ir—C27—C26	176.7 (3)
C4—N1—C3—C2	178.9 (3)	P—Ir—C27—C28	52.7 (6)
C1—N1—C4—C5	−119.8 (3)	C1—Ir—C27—C26	−98.8 (2)
C1—N1—C4—C6	117.1 (3)	C1—Ir—C27—C28	137.2 (3)
C3—N1—C4—C5	59.9 (4)	C26—Ir—C27—C28	−124.0 (3)
C3—N1—C4—C6	−63.1 (4)	C30—Ir—C27—C26	99.9 (2)
Ir—P—C8—C9	−122.0 (3)	C30—Ir—C27—C28	−24.1 (2)
Ir—P—C8—C13	63.8 (4)	C31—Ir—C27—C26	66.2 (2)
C8'—P—C8—C9	167 (2)	C31—Ir—C27—C28	−57.8 (2)
C8'—P—C8—C13	−7.4 (17)	Ir—C27—C28—C29	39.1 (4)
C14—P—C8—C9	4.9 (4)	C26—C27—C28—C29	−40.7 (4)
C14—P—C8—C13	−169.3 (3)	C27—C28—C29—C30	−34.5 (4)
C20—P—C8—C9	111.1 (3)	C28—C29—C30—Ir	12.4 (4)
C20—P—C8—C13	−63.1 (3)	C28—C29—C30—C31	95.2 (4)
P—C8—C9—C10	−173.9 (5)	P—Ir—C30—C29	−161.7 (2)
C13—C8—C9—C10	0.0	P—Ir—C30—C31	78.60 (17)
C8—C9—C10—C11	0.0	C1—Ir—C30—C29	−46.1 (4)
C9—C10—C11—C12	0.0	C1—Ir—C30—C31	−165.8 (2)
C10—C11—C12—C13	0.0	C26—Ir—C30—C29	43.0 (3)
C11—C12—C13—C8	0.0	C26—Ir—C30—C31	−76.72 (18)
P—C8—C13—C12	174.6 (4)	C27—Ir—C30—C29	6.8 (3)
C9—C8—C13—C12	0.0	C27—Ir—C30—C31	−112.94 (19)
Ir—P—C8'—C9'	−125.3 (3)	C31—Ir—C30—C29	119.7 (3)
Ir—P—C8'—C13'	58.0 (4)	Ir—C30—C31—C32	101.7 (3)
C8—P—C8'—C9'	−9.4 (16)	C29—C30—C31—Ir	−105.7 (3)
C8—P—C8'—C13'	174 (2)	C29—C30—C31—C32	−4.0 (5)
C14—P—C8'—C9'	9.8 (5)	P—Ir—C31—C30	−103.54 (17)
C14—P—C8'—C13'	−166.9 (3)	P—Ir—C31—C32	134.9 (2)
C20—P—C8'—C9'	116.8 (4)	C1—Ir—C31—C30	157.7 (3)
C20—P—C8'—C13'	−60.0 (4)	C1—Ir—C31—C32	36.1 (5)
P—C8'—C9'—C10'	−176.8 (5)	C26—Ir—C31—C30	99.28 (18)
C13'—C8'—C9'—C10'	0.0	C26—Ir—C31—C32	−22.3 (2)
C8'—C9'—C10'—C11'	0.0	C27—Ir—C31—C30	65.46 (18)
C9'—C10'—C11'—C12'	0.0	C27—Ir—C31—C32	−56.1 (2)
C10'—C11'—C12'—C13'	0.0	C30—Ir—C31—C32	−121.6 (3)
C11'—C12'—C13'—C8'	0.0	Ir—C31—C32—C33	33.6 (3)
P—C8'—C13'—C12'	176.7 (5)	C30—C31—C32—C33	−47.0 (4)
C9'—C8'—C13'—C12'	0.0	C31—C32—C33—C26	−27.6 (4)
Ir—P—C14—C15	58.5 (3)	Ir—C26—C33—C32	7.8 (3)
Ir—P—C14—C19	−121.2 (2)	C27—C26—C33—C32	91.4 (3)
C8—P—C14—C15	−72.2 (4)		
